# A Bovine Cell Line Resistant to Japanese Encephalitis Virus Entry but Permissive to Post-Entry Replication

**DOI:** 10.3390/v18020166

**Published:** 2026-01-27

**Authors:** Sang-Im Yun, Young-Min Lee

**Affiliations:** Department of Animal, Dairy, and Veterinary Sciences, College of Agriculture and Natural Resources, Utah State University, Logan, UT 84322, USA

**Keywords:** Japanese encephalitis virus, orthoflavivirus, flavivirus, infection, replication, entry, post-entry, susceptible, nonsusceptible

## Abstract

Japanese encephalitis virus (JEV) is a mosquito-borne zoonotic orthoflavivirus that poses a significant global health threat. It causes severe neuroinflammatory disease in humans and reproductive failure in swine. Because of the broad host range and cell tropism of JEV, identifying animal cell lines resistant to infection has been a persistent challenge. In this study, we demonstrate that Madin–Darby bovine kidney (MDBK) cells are resistant to JEV infection yet remain fully permissive to viral replication when transfected with viral genomic RNA. Using immunoblotting, immunofluorescence, and flow cytometry, we show that MDBK cells, unlike the highly susceptible baby hamster kidney (BHK-21) cells used as controls, do not support viral entry but sustain all post-entry stages of the replication cycle. Further investigation confirmed that MDBK cells possess a functional clathrin-mediated endocytic pathway, as evidenced by their susceptibility to bovine viral diarrhea virus, which relies on clathrin-dependent endocytosis for host cell entry. These findings establish MDBK cells as a nonsusceptible cell line for JEV entry despite intact endocytic function, providing a valuable platform for studying virus–host cell interactions and for identifying and validating host cell entry factors, a major challenge in JEV research.

## 1. Introduction

Japanese encephalitis virus (JEV) is a zoonotic orthoflavivirus within the *Flaviviridae* family, primarily transmitted by culicine mosquitos such as *Culex tritaeniorhynchus* [1]. Phylogenetically, JEV is closely related to other medically important mosquito-borne orthoflaviviruses, including West Nile virus, Zika virus, dengue virus, and yellow fever virus, as well as tick-borne orthoflaviviruses such as Powassan virus, Louping ill virus, and tick-borne encephalitis virus [2]. JEV causes symptomatic infections in both humans and livestock [3,4]. In humans, the virus can invade the central nervous system, leading to severe neuroinflammatory disease, most notably Japanese encephalitis (JE), which carries a case-fatality rate of ~15–25% [5,6]. Survivors often experience long-term neurological complications, including paralysis, cognitive deficits, speech difficulties, and behavioral disturbances [6,7]. Among livestock, swine are particularly vulnerable [8,9,10]. Infected pregnant sows may suffer reproductive disorders such as abortion, stillbirth, and fetal mummification, while newborn piglets can exhibit neurological symptoms including tremors and convulsions [9]. Boars may develop transient infertility due to testicular inflammation and reduced sperm quality [9,11]. These clinical manifestations, combined with JEV’s vector-borne and zoonotic nature, underscore its significance as a high-priority pathogen affecting both human and animal health [12].

Historically, JEV has been the leading cause of viral encephalitis across much of Asia and parts of the western Pacific [13,14]. However, its geographic range has expanded in recent years. Evidence of silent circulation has emerged in Europe, with JEV detected in birds and mosquitoes in Italy since the mid-1990s [15,16]. Additionally, a human case of asymptomatic JEV infection was reported in Angola in 2016, suggesting possible transmission in Africa [17]. To date, no autochthonous human cases of JE have been documented in either Europe or Africa. In the United States, JEV is increasingly regarded as an emerging public health concern, despite the absence of confirmed autochthonous cases in the Americas [18]. Currently, no antiviral treatments are available for JEV [19]. While both killed-inactivated and live-attenuated vaccines are used in endemic regions [20,21], JE continues to affect over 100,000 individuals annually across Asia [22]. The persistence of JE outbreaks, coupled with the ongoing expansion of JEV activity, highlights the urgent need for targeted antiviral therapies [23,24]. In this context, understanding viral replication is essential for identifying new molecular targets and developing novel therapeutic strategies [25].

JEV is an enveloped virus with a linear, positive-sense RNA genome of ~11 kb. The genome encodes a single polyprotein flanked by untranslated regions, which is cleaved into at least ten functional proteins [26]: three structural (capsid [C], pre-membrane [prM], and envelope [E]) and seven nonstructural (NS1, NS2A, NS2B, NS3, NS4A, NS4B, and NS5). The viral particle comprises an inner nucleocapsid formed by C proteins bound to the RNA genome [27], surrounded by a lipid bilayer embedded with prM/M and E proteins [28]. Infection begins with viral entry into host cells, mediated by the E protein, which facilitates receptor-mediated endocytosis and low pH-dependent fusion of viral and endosomal membranes, leading to uncoating and release of the viral genome into the cytoplasm [29,30]. The viral RNA is translated into a polyprotein, which is cleaved by host and viral proteases, including the NS2B–NS3 complex, into functional proteins [31]. A programmed -1 ribosomal frameshift in NS2A produces an extended NS1 protein, NS1′ [32,33]. RNA replication occurs in specialized membrane compartments derived from the endoplasmic reticulum (ER), where the replication complex, composed of NS3 and NS5, is assembled [34,35,36,37]. Newly synthesized genomes are packaged into nucleocapsids that bud into the ER, acquiring prM and E proteins to form immature virions [38,39,40,41]. These virions are transported through the secretory pathway and mature in the trans-Golgi network via prM cleavage by furin [42,43], resulting in infectious particles that are released from the cell [44]. While post-entry replication steps are relatively well characterized [45], the mechanisms underlying viral entry remain poorly understood [29,30].

Viral entry plays a pivotal role in determining host range and cell tropism, influencing the establishment, maintenance, and dissemination of infection [29,30]. Despite its importance, progress in understanding the molecular mechanisms underlying JEV entry has been limited, largely due to the absence of an animal cell line that selectively restricts viral entry while permitting downstream replication. Such a model system is essential for conducting genetic screens and functional studies of host factors involved in viral uptake. In this study, we identify a bovine cell line that is refractory to JEV entry yet fully supports all subsequent stages of the viral replication cycle. This finding addresses a critical gap in JEV research and provides a valuable platform for investigating the cellular components required for viral entry.

## 2. Materials and Methods

### 2.1. Cells and Viruses

Baby hamster kidney (BHK-21) cells were cultured in α-Minimum Essential Medium (α-MEM) supplemented with 10% heat-inactivated fetal bovine serum, 2 mM L-glutamine, 1× MEM vitamin solution, and 1× penicillin–streptomycin solution [46]. Madin–Darby bovine kidney (MDBK) cells were maintained in Dulbecco’s Modified Eagle Medium (DMEM) containing 10% heat-inactivated horse serum, 1 mM sodium pyruvate, and 1× penicillin–streptomycin solution [47]. Both cell lines were incubated at 37 °C in a humidified atmosphere with 5% CO_2_, and all media and reagents were obtained from Gibco (Carlsbad, CA, USA). A broader panel of animal cell lines was also used for the screening experiments, and these are described in the Results section. The viruses used were the pathogenic JEV strain CNU/LP2, derived from a full-length infectious cDNA clone (pBAC^SP6^/JVFL) [48,49], and the cytopathic bovine viral diarrhea virus (BVDV) strain NADL, derived from a full-length infectious cDNA clone (pACNR/NADL) [47,50].

### 2.2. Immunoblotting

Immunoblotting was performed as previously described [31]. Briefly, cells cultured in 6-well plates were lysed in 200 μL of sample loading buffer containing 80 mM Tris-HCl (pH 6.8), 2% sodium dodecyl sulfate (SDS), 10% glycerol, 100 mM dithiothreitol, and 0.2% bromophenol blue. Lysates were boiled for 5 min, separated by SDS–polyacrylamide gel electrophoresis, and transferred to polyvinylidene difluoride membranes. Membranes were blocked with 5% nonfat dry milk in phosphate-buffered saline (PBS) containing 0.2% Tween 20, then incubated with primary antibodies (1:1000): mouse anti-JEV hyperimmune antiserum (American Type Culture Collection, Manassas, VA, USA), rabbit anti-JEV C antiserum [31], or rabbit anti-JEV NS1 antiserum [31]. After washing, membranes were incubated with alkaline phosphatase (AP)-conjugated goat anti-mouse or anti-rabbit IgG secondary antibodies (1:5000; Jackson ImmunoResearch, West Grove, PA, USA). Immunoreactive bands were visualized using AP substrates: 5-bromo-4-chloro-3-indolyl phosphate and nitroblue tetrazolium (Sigma-Aldrich, St. Louis, MO, USA).

### 2.3. Immunofluorescence Assay

Immunofluorescence assays were performed as described [48,51]. Cells grown in 4-well chamber slides were fixed and permeabilized with cold methanol for 10 min, followed by three 10 min washes with PBS. Blocking was performed in PBS containing 5% bovine serum albumin (BSA) for 30 min, followed by three PBS washes. Cells were incubated for 2 h with mouse anti-JEV hyperimmune antiserum (1:1000 in PBS with 2.5% BSA; American Type Culture Collection), washed three times with PBS, and then incubated for 2 h with Alexa Fluor 488-conjugated goat anti-mouse IgG (1:1000 in PBS with 2.5% BSA; Molecular Probes, Eugene, OR, USA). For nuclear staining, cells were incubated for 30 min with FxCycle PI/RNase staining solution (Molecular Probes), washed twice with PBS, and mounted using antifade medium (Dako, Glostrup, Denmark). Images were acquired using an LSM-710 confocal microscope (Carl Zeiss, Jena, Germany).

### 2.4. Flow Cytometry

Flow cytometry was performed as previously described [52]. Cells cultured in 6-well plates were harvested by trypsinization, washed with PBS, and pelleted by centrifugation. Pellets were fixed in Cytofix/Cytoperm solution (BD Biosciences, San Jose, CA, USA) for 20 min at 4 °C. Subsequent steps were carried out in Perm/Wash buffer (BD Biosciences). Fixed cells were washed twice, incubated with mouse anti-JEV hyperimmune antiserum (1:500; American Type Culture Collection) for 1 h at 4 °C, washed again, and then incubated with Alexa Fluor 488-conjugated goat anti-mouse IgG (1:1000; Molecular Probes) for 1 h at 4 °C. After final washes, cells were resuspended in Perm/Wash buffer and analyzed using a FACSAria III cell sorter with Diva 6.1.3 software (BD Biosciences), acquiring 20,000 events per sample.

### 2.5. Viral Growth and Plaque Morphology

The growth of BVDV in MDBK cells was evaluated as described [47]. MDBK cells were seeded in 60 mm dishes overnight and infected with BVDV at a multiplicity of infection (MOI) of 1 for 1 h, with agitation every 10 min. After infection, the cells were washed once with DMEM and incubated in complete medium for 3 days at 37 °C with 5% CO_2_. Supernatants were collected at 6, 12, 24, 36, 48, and 60 h after infection for virus titration by plaque assay. For plaque assays, MDBK cells were seeded in 6-well plates at 5 × 10^5^ cells/well overnight and infected with 10-fold serial dilutions of the supernatants for 1 h, with agitation every 10 min. After infection, the inocula were removed, and the cells were overlaid with 3 mL/well of MEM containing 5% heat-inactivated horse serum and 0.5% SeaKem LE agarose (FMC BioProducts, Rockland, ME, USA), then incubated for 4 days at 37 °C with 5% CO_2_. After incubation, the overlays were removed, and the cells were fixed with 7% formaldehyde and stained with 1% crystal violet in 5% ethanol.

## 3. Results

We screened a diverse panel of animal cell lines representing multiple species to identify a cell line resistant to JEV entry yet capable of supporting all post-entry stages of the viral replication cycle (Table 1). Although multiple screening attempts failed to yield a suitable candidate, we ultimately identified the bovine MDBK cell line, which is not susceptible to JEV entry but is permissive to viral RNA replication and infectious particle production.

### 3.1. Identification of a JEV-Resistant Bovine Cell Line, MDBK, via Whole-Cell Lysate Analysis

**Figure 1:**
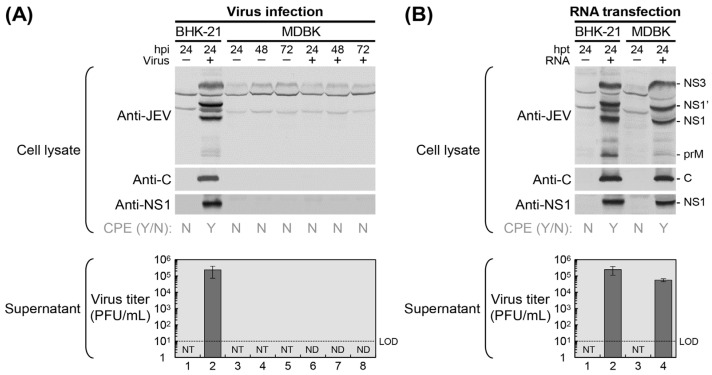
MDBK cells resist JEV infection but support intracellular viral RNA replication and virus production. MDBK and BHK-21 cells were either mock-infected or infected with JEV strain CNU/LP2 at an MOI of 1 (**A**), or mock-transfected or transfected with 1 μg of in vitro-transcribed RNA derived from a full-length infectious cDNA clone of JEV strain CNU/LP2 (**B**). Infection was performed for 1 h, followed by two washes with complete medium. At the indicated time points, cell lysates were analyzed by immunoblotting using a mouse anti-JEV hyperimmune antiserum (Anti-JEV) or rabbit polyclonal antibodies specific for the JEV C (Anti-C) and NS1 (Anti-NS1) proteins. Cytopathic effects (CPE) were evaluated by phase-contrast microscopy and recorded as present (Y) or absent (N). Culture supernatants were collected for virus titration by plaque assay on BHK-21 cells. hpi, hours post-infection; hpt, hours post-transfection; LOD, limit of detection; NT, not tested; ND, not detected.

To assess the susceptibility of MDBK cells to JEV infection, we performed immunoblot analysis using whole-cell lysates following infection with viral particles (Figure 1A). MDBK cells were either mock-infected or infected with the highly pathogenic JEV strain CNU/LP2 [48,49] at an MOI of 1. Total cell lysates were harvested at 24, 48, and 72 h post-infection (hpi) and probed for the accumulation of viral proteins, including the structural protein C and the nonstructural protein NS1. Immunoblotting with a mouse anti-JEV hyperimmune antiserum, along with two rabbit antisera specific to JEV C and NS1 proteins, revealed no detectable expression of viral proteins in JEV-infected MDBK cells at any of the three time points. In contrast, JEV-infected BHK-21 cells, used as a positive control [48], showed strong expression of viral proteins as early as 24 hpi, confirming their high susceptibility to JEV infection. Consistent with the absence of viral protein synthesis, no cytopathic effects (CPE) were observed in MDBK cells, regardless of infection status, throughout the entire 72 h observation period. As expected, JEV-infected BHK-21 cells exhibited pronounced CPE, while mock-infected counterparts remained unaffected. Furthermore, supernatants from both JEV-infected and mock-infected MDBK cells contained no detectable infectious virus particles at any of the three time points up to 72 hpi, further supporting the lack of productive infection. In contrast, supernatants from JEV-infected BHK-21 cells yielded a high viral titer, reaching ~2.4 × 10^5^ plaque-forming units per mL (PFU/mL) as early as 24 hpi.

To evaluate whether MDBK cells support JEV RNA replication when the viral entry step is bypassed, we transfected the cells with 1 μg of infectious RNA transcribed in vitro from a full-length cDNA clone of JEV strain CNU/LP2 (Figure 1B). In parallel, BHK-21 cells were used as a positive control. At 24 h post-transfection (hpt), immunoblot analysis using the same panel of JEV-specific antisera revealed high levels of viral protein accumulation in RNA-transfected MDBK cells, nearly comparable to those observed in RNA-transfected BHK-21 cells. As expected, no viral proteins were detected in mock-transfected MDBK or BHK-21 cells. In agreement with these findings, RNA-transfected MDBK and BHK-21 cells both exhibited strong CPE at 24 hpt, whereas mock-transfected cells remained morphologically normal. Notably, the visible characteristics of CPE differed between the two cell lines: BHK-21 cells displayed membrane blebbing, while MDBK cells exhibited perinuclear vacuole formation. Viral RNA replication in MDBK cells was further confirmed by the production of infectious virions in the supernatant, with titers reaching ~5.6 × 10^4^ PFU/mL at 24 hpt, slightly lower than the titer of ~2.5 × 10^5^ PFU/mL detected in the supernatant from BHK-21 cells at the same time point. Collectively, these results indicate that MDBK cells are not susceptible to JEV entry but are fully permissive to all subsequent stages of the viral replication cycle.

### 3.2. Validation of JEV Resistance in MDBK Cells Using Single-Cell-Based Assays

**Figure 2:**
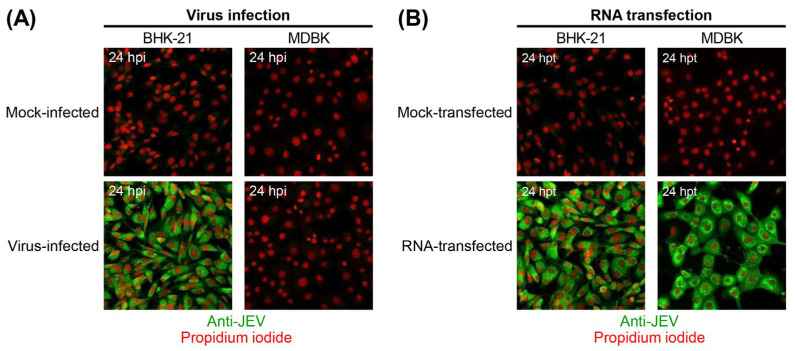
MDBK cells are resistant to JEV infection but permissive to viral RNA replication. MDBK and BHK-21 cells were mock-infected or infected with JEV strain CNU/LP2 at an MOI of 5 (**A**), or mock-transfected or transfected with 5 μg of in vitro-transcribed RNA from a full-length infectious cDNA clone of JEV strain CNU/LP2 (**B**). At 24 h post-infection (hpi) or post-transfection (hpt), cells were fixed and subjected to IFA using a mouse anti-JEV hyperimmune antiserum (Anti-JEV) and Alexa Fluor 488-conjugated goat anti-mouse IgG. Nuclei were counterstained with propidium iodide. Immunoreactive proteins were visualized by confocal microscopy.

To validate our observation that MDBK cells are resistant to JEV entry but permissive to viral RNA replication, we performed immunofluorescence assays (IFA) to detect viral protein expression in individual cells following either virus infection or RNA transfection (Figure 2). MDBK cells were mock-infected or infected with JEV strain CNU/LP2 at an MOI of 5, or mock-transfected or transfected with 5 μg of infectious RNA synthesized in vitro from a full-length cDNA clone of the same JEV strain. BHK-21 cells, known to be highly susceptible to JEV, served as a positive control. At 24 hpi or hpt, cells were immunostained with a mouse anti-JEV hyperimmune antiserum and examined by confocal microscopy. As expected, all virus-infected BHK-21 cells were JEV-positive, whereas virus-infected MDBK cells lacked detectable JEV antigens (Figure 2A). In contrast, both RNA-transfected MDBK and BHK-21 cells were JEV-positive (Figure 2B). These results confirm that MDBK cells are not susceptible to JEV entry but are fully permissive to viral RNA replication.

**Figure 3:**
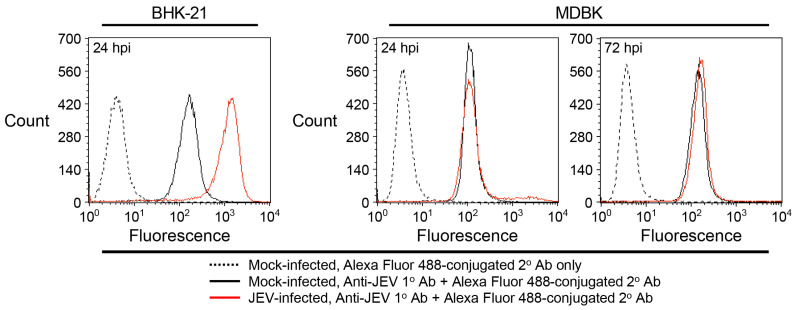
MDBK cells are not susceptible to JEV infection. MDBK and BHK-21 cells were mock-infected or infected with JEV strain CNU/LP2 at an MOI of 5. At the indicated time points, cells were fixed and stained with a mouse anti-JEV hyperimmune antiserum, followed by Alexa Fluor 488-conjugated goat anti-mouse IgG. Mock-infected cells stained only with the secondary antibody served as a negative control to establish the fluorescence baseline. JEV-positive cells were quantified by flow cytometry. hpi, hours post-infection.

To further support our interpretation that MDBK cells are nonsusceptible to JEV entry, we performed flow cytometry following JEV infection. MDBK cells were mock-infected or infected with JEV strain CNU/LP2 at an MOI of 5, with BHK-21 cells again serving as a positive control. At 24 hpi, cells were immunostained with the mouse anti-JEV hyperimmune antiserum, and JEV-positive cells were counted by flow cytometry. The results were consistent with the IFA findings: nearly all virus-infected BHK-21 cells were JEV-positive at 24 hpi, whereas virus-infected MDBK cells remained JEV-negative at that time point, and this status persisted through 72 hpi (Figure 3). These findings reinforce the conclusion that MDBK cells are resistant to JEV infection.

### 3.3. Assessment of Clathrin-Mediated Endocytic Pathway Functionality in MDBK Cells

**Figure 4:**
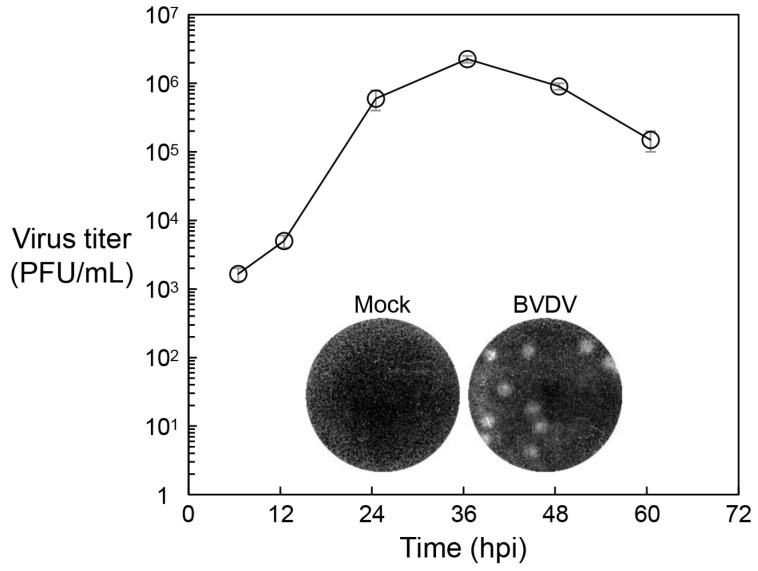
MDBK cells are highly susceptible to BVDV infection. MDBK cells were infected with BVDV strain NADL at an MOI of 1. At the indicated time points, culture supernatants were collected, and virus titers were determined by plaque assay on MDBK cells. Titers are expressed as plaque-forming units per mL (PFU/mL). Virus titers represent the mean ± standard deviation from two biological replicates. Representative plaques, visualized by crystal violet staining, are shown within the graph. hpi, hours post-infection.

Because JEV enters non-neuronal host cells through the clathrin-mediated endocytic pathway [29,30,53], we evaluated the functionality of this pathway in MDBK cells by examining their susceptibility to BVDV, an enveloped positive-strand RNA virus that also depends on clathrin-dependent endocytosis [54,55,56]. MDBK cells were infected with the cytopathic BVDV strain NADL [47,50] at an MOI of 1, and culture supernatants were collected at 6, 12, 24, 36, 48, and 60 hpi to assess viral growth kinetics via plaque assays. In contrast to their resistance to JEV infection, MDBK cells were highly susceptible to BVDV, exhibiting robust viral replication and the formation of clearly visible plaques (Figure 4). Consistent with these observations, inhibitors of clathrin-mediated uptake (e.g., chlorpromazine, dominant-negative EPS15, and dominant-negative dynamin), as well as inhibitors of endosomal acidification have been shown to block BVDV infection in MDBK cells [54,55]. Similar results in fetal bovine kidney cells, including co-localization of BVDV with clathrin, EEA1, and LAMP2, further support that BVDV enters host cells via clathrin-coated pits followed by low-pH-dependent fusion in endosomes [56]. Together, these findings demonstrate that the clathrin-mediated endocytic machinery is fully functional in MDBK cells.

## 4. Discussion

Although decades of research have clarified many aspects of the JEV replication cycle, the molecular mechanisms of viral entry remain incompletely understood [29,30]. This knowledge gap largely reflects the broad host range and cell tropism of the virus [57], which complicate the development of models that clearly distinguish nonsusceptible from susceptible cells. A cell line that restricts viral entry while supporting post-entry replication would provide a powerful tool for studying cellular determinants of viral uptake. To address this need, we identified and characterized MDBK cells, which are not susceptible to JEV entry but are fully permissive to subsequent replication steps. Here, we report this selective phenotype and provide a comprehensive analysis of cell susceptibility to JEV infection. Notably, immunoblotting, immunofluorescence, and flow cytometry all demonstrate that MDBK cells exhibit a defect in viral entry despite intact endocytic function, while permitting all post-entry steps of viral replication. This nonsusceptible cell model offers a foundation for investigating virus–host cell interactions, particularly for identifying and validating host cell entry factors [58], which are critical for understanding JEV pathogenesis and for designing antiviral strategies targeting early stages of infection.

Previous studies examining numerous cell lines derived from multiple animal species (e.g., human, monkey, pig, rabbit, mouse, rat, hamster, and insect) have demonstrated substantial variability in susceptibility to JEV infection, with several notable observations [59,60,61,62,63,64,65,66,67]: (i) a mutant BHK-21 derivative generated by DNA alkylation exhibited markedly reduced viral binding compared with its parental cell line (~2% vs. ~49%) and showed decreased expression of membrane proteins, including annexin A1/A2 and voltage-dependent anion channel 1/2 [59]; (ii) rat kidney-derived NRK cells displayed lower viral binding than monkey kidney-derived Vero cells, implicating a 74 kDa membrane protein as a potential host cell entry factor [60]; (iii) differentiated rat neuronal CSM14.1 cells exhibited reduced viral entry and RNA replication relative to undifferentiated counterparts, with the latter reduction associated with an enhanced interferon response [61]; and (iv) persistent infection has been reported in mammalian (e.g., rabbit kidney-derived MA-111) and insect (e.g., *Spodoptera frugiperda*-derived Sf9) cell lines, characterized by low-level virus release and resistance to superinfection [62,63,64]. To date, no continuous cell line has been identified as completely resistant to JEV infection. These previous studies underscore the complexity of defining host-specific entry factors and highlight the need for cell models that selectively restrict viral entry while supporting post-entry replication, reinforcing the significance of our present study.

Pigs and cattle are considered two of the most important livestock species. Of these, pigs are highly susceptible to JEV infection and serve as major virus-amplifying hosts in endemic regions [13,14], producing high viremia yet relatively mild clinical disease that enables mosquito-mediated transmission to humans [68,69,70,71,72,73]. In experimentally infected pigs, a variety of organs and tissues, including lymph nodes, spleen, thymus, kidney, liver, and brain, particularly tonsils, support robust JEV replication, consistent with the epidemiological role of pigs in sustaining viral circulation [74,75]. In contrast, cattle exhibit markedly lower susceptibility and are regarded as dead-end hosts [4,14], because they produce low viremia insufficient to infect mosquitoes and show limited evidence of productive infection [76,77,78]. Intriguingly, bovine resistance may reflect differences in receptor availability or innate antiviral factors, such as bovine lactoferrin, which inhibits JEV entry, presumably by interacting with heparan sulfate and low-density lipoprotein receptor-related proteins [79]. Serological surveys further support this disparity between pigs and cattle, with pigs exhibiting high seroprevalence, whereas cattle rarely show seropositivity [80,81,82]. By providing a cell model that restricts entry yet permits post-entry replication, our study offers a unique platform to investigate cellular components required for JEV entry. For example, screening JEV entry in nonsusceptible cells such as MDBK using cellular genes derived from susceptible cells such as neurons could identify key virus–host cell interactions involved in JEV internalization, ultimately guiding the design of entry inhibitors targeting the earliest steps of infection.

While MDBK cells provide a unique and powerful system for investigating host determinants of JEV entry, several limitations of the present study should be acknowledged. Most importantly, although our findings establish that MDBK cells restrict JEV at the entry step while supporting all subsequent stages of the viral life cycle, the specific cellular components responsible for this restriction remain undefined. Mechanistic studies addressing questions such as whether MDBK cells lack specific surface molecules required for JEV entry, or whether overexpression of candidate host factors can restore susceptibility, are scientifically valuable but fall beyond the scope of the current work. Additional biochemical or visualization-based analyses related to viral RNA replication and virion release in MDBK cells would further substantiate our conclusion that these cells support post-entry replication. Furthermore, the resistance phenotype observed in MDBK cells may reflect species-specific features that do not fully reproduce viral entry mechanisms in natural hosts, and other cellular properties, including membrane composition or signaling pathways, may also contribute to susceptibility. Addressing these limitations will require systematic identification of candidate entry factors, functional validation of their roles in MDBK cells, and comparative analyses across diverse host species to clarify the broader relevance of the observed restriction.

## Figures and Tables

**Table 1:** Cells screened in this study.

Organism	Cell	Origin
Human	HEK-293	Embryonic kidney
Human	HeLa	Cervix
Human	HepG-2	Liver
Human	Huh-7	Liver
Human	MOLT-4	Peripheral blood (T lymphoblast-like)
Human	SH-SY5Y	Bone marrow (neuroblast-like)
Monkey	Vero	Kidney
Monkey	MARC-145	Kidney
Pig	ST	Testis
Pig	PK-15	Kidney
Cow	MDBK	Kidney
Horse	NBL-6	Skin dermis
Sheep	SFF-6	Fetus (fibroblast)
Goat	GFF-4	Fetus (fibroblast)
Dog	MDCK	Kidney
Cat	CRFK	Kidney
Mouse	MEF	Embryonic fibroblast (C57BL/6 strain)
Mouse	NIH/3T3	Embryonic fibroblast (NIH/Swiss strain)
Mouse	NSC-34	Spinal cord (motor neuron-like)
Mouse	Neuro-2a	Neural crest (neuroblast-like)
Hamster	BHK-21	Kidney
Chicken	CEF	Embryonic fibroblast
Mosquito	C6/36	Larva (*Aedes albopictus*)

## Data Availability

All data generated in this study are included in this manuscript or are available upon request from the authors.
